# A new method for quantitative assessment of hand muscle volume and fat in magnetic resonance images

**DOI:** 10.1186/s41927-020-00170-3

**Published:** 2020-12-22

**Authors:** Andreas Friedberger, Camille Figueiredo, Tobias Bäuerle, Georg Schett, Klaus Engelke

**Affiliations:** 1 Institute of Medical Physics, University of Erlangen-Nuremberg, Henkestraße 91, 91052 Erlangen, Germany; 2 Department of Medicine 3, University of Erlangen-Nuremberg, Erlangen, Germany; 3 Radiological Institute, University Hospital of Erlangen-Nuremberg, Erlangen, Germany

**Keywords:** Random forest segmentation, Hand muscle, Fat quantification, Rheumatoid arthritis

## Abstract

**Background:**

Rheumatoid arthritis (RA) is characterized by systemic inflammation and bone and muscle loss. Recent research showed that obesity facilitates inflammation, but it is unknown if obesity also increases the risk or severity of RA. Further research requires an accurate quantification of muscle volume and fat content.

**Methods:**

The aim was to develop a reproducible (semi) automated method for hand muscle segmentation and quantification of hand muscle fat content and to reduce the time consuming efforts of manual segmentation. T1 weighted scans were used for muscle segmentation based on a random forest classifier. Optimal segmentation parameters were determined by cross validation with 30 manually segmented hand datasets (gold standard). An operator reviewed the automatically created segmentation and applied corrections if necessary. For fat quantification, the segmentation masks were automatically transferred to MRI Dixon sequences by rigid registration. In total 76 datasets from RA patients were analyzed. Accuracy was validated against the manual gold standard segmentations.

**Results:**

Average analysis time per dataset was 10 min, more than 10 times faster compared to manual outlining. All 76 datasets could be analyzed and were accurate as judged by a clinical expert. 69 datasets needed minor manual segmentation corrections. Segmentation accuracy compared to the gold standard (Dice ratio 0.98 ± 0.04, average surface distance 0.04 ± 0.10 mm) and reanalysis precision were excellent. Intra- and inter-operator precision errors were below 0.3% (muscle) and 0.7% (fat). Average Hausdorff distances were higher (1.09 mm), but high values originated from a shift of the analysis VOI by one voxel in scan direction.

**Conclusions:**

We presented a novel semi-automated method for quantitative assessment of hand muscles with excellent accuracy and operator precision, which highly reduced a traditional manual segmentation effort. This method may greatly facilitate further MRI image based muscle research of the hands.

## Background

Rheumatoid arthritis (RA) is a chronic disease characterized by inflammation of the synovial membrane (synovitis) and loss of bone, cartilage and muscle [1]. In contrast to bone, muscle is not yet in the spotlight of RA research, although studies show that muscle atrophy is a hall mark of RA [2, 3]. Compared to healthy controls, RA patients have an accelerated loss of fat-free skeletal muscle, so called rheumatoid cachexia [4] and impaired hand motor performance [5], probably caused by muscle fiber degeneration.

It is well known that obesity is associated with systemic inflammation, because adipose tissue is a source of pro-inflammatory cytokines and triggers inflammatory responses [6, 7]. In patients with RA, muscle fat infiltration of the thigh contributes to low physical function and activity [8]. Another study reported lower calf muscle area and density in RA patients compared to controls and also found associations between greater joint destruction and greater muscle deficits. Whether these findings can be explained by a cause-effect relation between muscle fat infiltration and RA is a pending question [9], as so far the interrelationships of inflammation and muscle dysfunction [10] in RA is poorly understood.

Obviously, the quantification of muscle volume and fat content is the first step to address this question. The hand with its multitude of joints and associated synovial fluids is usually most severely affected by RA and is probably the prime target for the investigation of RA related muscle and fat characteristics. However, the small volume and multiple different tissues like bone, tendons, vessels and muscle require a sophisticated segmentation for the quantification of muscle volume and adipose tissue in the hand.

Manual segmentation is tedious and for complicated tasks can take hours. In contrast, a fully automated segmentation may be desirable to supersede the need for manual interactions, but in our experience rarely works in elderly subjects, in which pathological conditions often define most of the population variance. In the hand many small and rapidly changing anatomical structures further complicate a segmentation. In particular, in clinical studies, a careful review of any automatic segmentation process with the options of manual editing by experts is typically required. Thus, in this paper we describe a method, which provides an automated initial segmentation, with two aims:

- To significantly reduce overall processing time compared to a standard manual slice by slice based segmentation approach
- To provide manual editing, but to limit these interactions to a minimum in order to improve reanalysis precision.

The core of the automated process is based on a random forest algorithm running on standard clinical T1 weighted magnetic resonance (MR) images. It exploits the characteristic muscle feature of a distinct grey value throughout the acquired stack of MR images. 3D editing after the automatic segmentation part can be performed using a set of tools developed earlier [11]. Fat quantification is then done using 2-pt Dixon (MR) images of the hand.

To the best of our knowledge such a method for hand muscle segmentation has not been published yet. So far studies on muscle and fat quantification mainly targeted the thigh, where simple threshold based techniques to separate subcutaneous adipose tissue (SAT), muscle and the femoral bone worked well [12]. A more advanced approach used a combination of fuzzy clustering, morphological operations and active snakes to segment the deep fascia lata of the thigh muscles [13] to further differentiate SAT from intermuscular adipose tissue. Another study [14] used a random walk graph-based formulation with incorporated prior knowledge of shape to segment the individual muscles of the thigh.

## Methods

### Patient details

MR hand scans of 76 outpatients with RA (37 males, 39 females), were acquired at the Rheumatology Outpatient Clinic of the University of Erlangen. Apart from the diagnosis of RA, no other inclusion or exclusion criteria were applied. Patient characteristics are shown in Table 1.

**Table 1:** Patient characteristics

*n* = 76	Mean	SD	Min	Max
Age [y]	61	14	26	87
Disease duration [y]	8	7	1	34
BMI	25.6	4.2	17.5	36.7
DAS28	2.7	1.2	0.3	5.6
ESR [mm/hr]	17	16	1	71
CRP [mg/L]	6	11	0	59

*Abbreviations*: *SD* standard deviation, *BMI* body mass index, *CRP* C-reactive protein, *DAS28* Eular disease activity score, *ESR* erythrocyte sedimentation rate

### MRI scans

This study utilized two different MR sequences – a T1 weighted scan and a two-point Dixon scan, described in detail below. These two sequences were part of the standard hand protocol of the Rheumatology Outpatient Clinic of the University of Erlangen. The scans and the protocol were not tailored to this study, but rather part of clinical routine and were typically performed once annually. The scans were performed on the dominant hand of the patient. The patient consent form included the agreement to also use these data for research purposes (Ethics approval 52_14B of the Medical Faculty of FAU Erlangen-Nuremberg).

MR imaging was performed on a 1.5-Tesla MR system (MAGNETOM Aera, Siemens Healthcare GmbH, Erlangen, Germany) with a hand/wrist radio frequency 16-channel coil. Subjects were positioned prone with head first.

The protocol consisted of a standard axial fat saturated T_1w_ turbo spin echo (TSE) sequence (Matrix size: 320 × 320, Voxel size: 0.5 × 0.5 × 3.0 mm^3^, Slices: 64, TR: 760 ms, TE: 13 ms) and an axial T_2w_ two point Dixon TSE sequence (Matrix size: 320 × 320, Voxel size: 0.5 × 0.5 × 3.0 mm^3^, Slices: 30, TR: 3040 ms, TE: 78 ms, TD: 0 ms). Throughout this paper, these two sequences are referred to as T1 weighted and Dixon sequences.

**Fig. 1:**
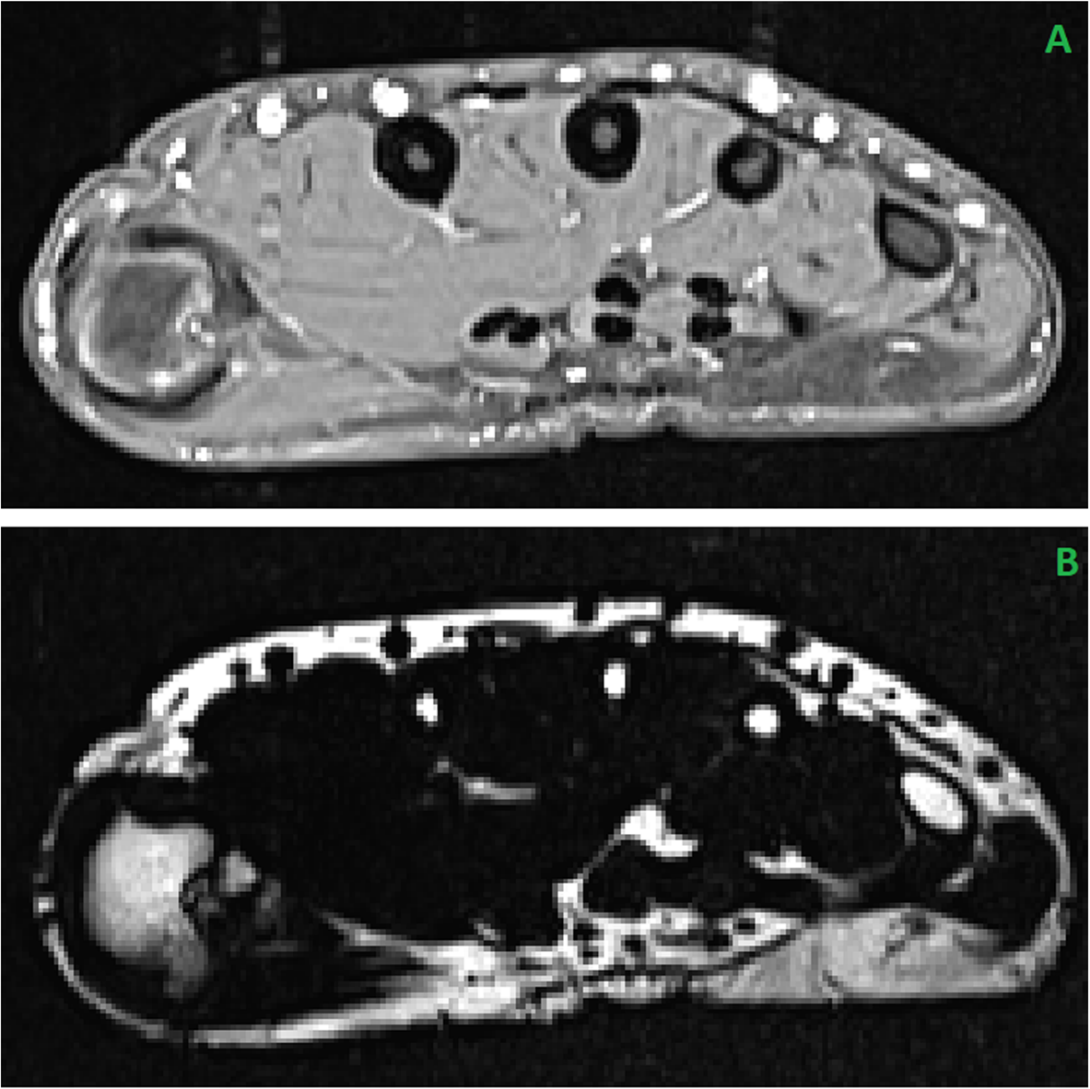
**a** Transversal slice of a T1 weighted fat suppressed MRI hand scan in the metacarpal region. **b** Corresponding slice of a quantitative Dixon fat fraction image. The grey value of each voxel corresponds to a fat ratio, where 1 grey value equals 0.1%

The Dixon sequence produces a fat and water images [15] calculated from two acquired spin echo images - one with water and fat signals in phase, the other out of phase. These two images are used to calculate the pure-water V_water_ and pure-fat images V_fat_. Applying equation

1 $$ {V}_{ff}=\frac{V_{fat}}{V_{water}+{V}_{fat}}\ast 1000{\mbox{\fontencoding{U}\fontfamily{wasy}\selectfont\char104}} $$

results in a quantitative fat fraction (FF) image. This image assigns the percentage of fat (Fig. 1b) to every voxel. The fat fraction intensity values range from 0 to 1000, which corresponds to 0% to 1000‰ fat per voxel.

### Image processing overview

Muscle segmentation was performed in the T1 weighted scans (Fig. 1a), because the muscle boundary is difficult to detect in Dixon fat images (Fig. 1b). The main component of the automated segmentation was a random forest classifier.

**Fig. 2:**
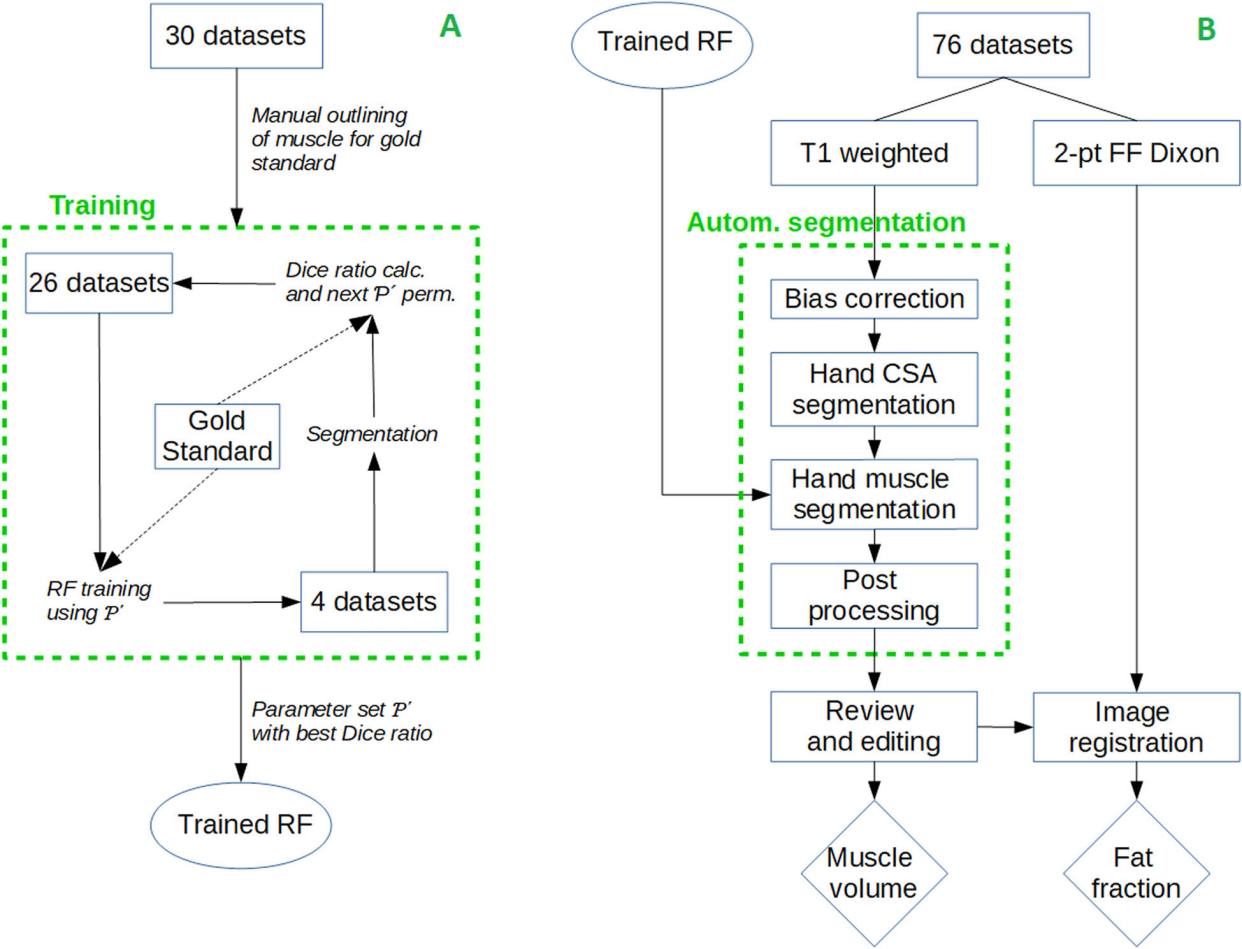
Overview of the RF training (**a**) on 30 randomly chosen datasets from the cohort, and of the segmentation workflow on all 76 datasets, utilizing the trained RF (**b**)

It was trained on 30 randomly chosen datasets, which had been segmented manually by a medical expert (gold standard). The trained RF was subsequently used to segment muscle in all 76 datasets. These results were again reviewed by a clinical expert and edited as necessary. The 30 gold standard datasets were used for the validation of accuracy. 14 of the 76 datasets were used for precision analysis. The two distinct domains of the method are depicted in Fig. 2 and consist of the following:

1. Random forest training
2. Segmentation workflow

- Pre processing of T1 scans to remove image inhomogeneities
- Segmentation of hand cross sectional area (CSA)
- Random forest segmentation of hand muscle
- Rigid multimodal registration of segmentation mask to Dixon fat image

Random forest training is required once only and learns the RF to detect muscle. The following sub chapters describe these steps in detail.

### Random forest training

Random forest (RF) is a well-known ensemble learning method from machine learning [16], but is also widely used for image segmentation [17–19]. Before being able to used it for classification, it must be trained on training data.

A RF consists of an ensemble of decision trees of an arbitrary but set number and are trained for a specific problem using training data. The RF input are features, which are calculated for each voxel. Usually a set of different features is created for each voxel. The RF output are labels, which are ‘muscle’ and ‘background’ in our study. Background in our case is everything except muscle, i.e. air but also soft tissue, bone, tendons etc. During the RF training, features and their corresponding labels have to be provided, so the RF is able to learn its decisions. Randomness is introduced by picking a random subset of the available feature-label pairs and a random subset of features for each decision node of each tree. The determination of the optimal RF parameter set (Ƥ_RF_), i.e. the number of trees, the maximal tree depth and the number of samples per decision node, was part of the training. The number of used features per node was empirically set to the square root of the number of features as typically used for RF.

#### Feature description

Features are the image ‘properties’ based on which the RF makes its decision. In this study the following features were used and calculated for each voxel of the T1 image:

- Mean grey value and standard deviation of 2D (in-plane) neighborhoods
- Mean grey value and standard deviation of 3D neighborhoods
- Gradient magnitude by Sobel operator
- Extended Local Binary Pattern
- Gabor filter

Mean grey values and standard deviations were calculated in voxel neighborhoods with different radii. Since the ratio between in-plane voxel size and slice thickness was 6, 2D and 3D neighborhoods were differentiated. In the 2D case, only neighboring voxels in the slice itself were considered; voxel radii were 1, 2 and 3 in city block distance. In the 3D case, voxels of the two adjacent slices were taken into account, too. In-plane voxel radii were 4, 5 and 6 in city block distance.

**Fig. 3:**
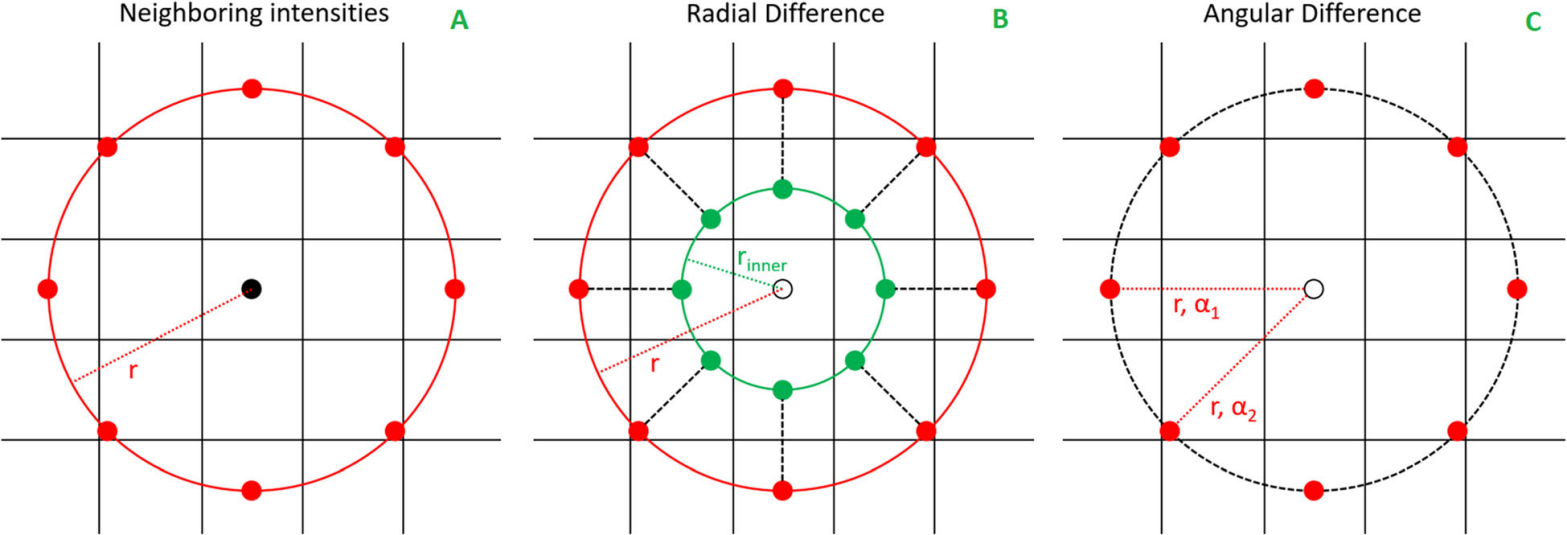
The in-plane sample points of three of the four 2D extended local binary pattern. **a** Intensity difference between the mean of the neighboring sample points on a circle (red) and the central voxel. **b** Intensity difference between sample points with two different radii. **c** Intensity difference between sample points with same radius but different angle

Extended Local Binary Pattern (ELBP) are 2D texture descriptors calculated for each slice individually. They are invariant to monotonic intensity changes and require little processing time [20]. Two specific ELBP pattern encode intensity relationships of the grey value of a voxel with the mean grey value of the image (ELBP_CI) and of a neighborhood with a specific radius *r* around the voxel (ELBP_NI) (Fig. 3). Two other pattern encode radial (ELBP_RD) and angular (ELBP_AD) grey value differences around the voxel. These two radial pattern were calculated using sampling in polar coordinates around the center voxel (Fig. 3). Sample points S were characterized by the radius *r* and an angle *α*, S=S(*r*, *α*). The angle was determined by the number of samples, which were arranged equidistantly on a circle with radius *r*. The sample grey values were linearly interpolated. Radial differences (RD) were calculated from sample pairs with same angle but different radii, S(*r*_*1*_, *α*_*n*_) and S(*r*_*2*_, *α*_*n*_) and angular differences (AD) from samples pairs with same radius but different angles, S(*r*, *α*_*n*_) and S(*r*, *α*_*n + 1*_). The determination of the optimal ELBP parameter set (Ƥ_ELBP_), i.e. the number of samples and the circle radii, was part of the training and described below.

The Gabor filter response for each voxel was calculated from an in-plane kernel around the voxel. In this area, the image grey values were modulated by a 2D sinusoidal wave and convoluted by a Gaussian function. In our case the sinusoidal was rotated in-plane by 0°, 45°, 90° and 135°, resulting in 4 different Gabor filter outputs. These were applied to each slice individually. As for ELBP, the optimal Gabor filter parameter set (Ƥ_GF_), i.e. the Gaussian σ, the sinusoidal wavelength (λ) and the kernel size, was determined by the training.

#### Training

The training workflow is depicted in Fig. 2a. Aim of the RF training is the determination of the optimal parameter set Ƥ, as the union of the above mentioned parameter sets: Ƥ = Ƥ_RF_ ∩ Ƥ_GF_ ∩ Ƥ_ELBP_. For the training, 30 datasets were randomly selected from the cohort for which a clinical expert manually outlined the hand muscle to provide the correct labels used as gold standard. Since intensities of the same tissue differ among MR scans (even if obtained from the same scanner and corrected by N4ITK, see below), the T1 scans had to be normalized for comparable features across datasets. This normalization was performed according to Eq. (2), where V and V_norm_ were the original and normalized voxel grey values, and V_muscle_ and V_cort_ were the grey values of the peak maxima of cortical bone and muscle in the grey value histogram, respectively:

2 $$ {V}_{norm}=\frac{V-{V}_{cort}}{V_{muscle}-{V}_{cort}}\ast 100 $$

The normalized distance between V_muscle_ and V_cort_ was chosen to be 100 intensity units. V_muscle_ and V_cort_ were obtained by a watershed-like thresholding of the histogram values, until the two maxima were left. The features described in the prior section were calculated using these normalized grey value images.

Ƥ was determined by an iterative process involving 7-fold cross-validation (CV-7). For this purpose, the 30 datasets were split into two subsets with a 6:1 cardinality ratio: a training set of 26 (≈30/7) and a validation set of 4 datasets. Initially, a sensible value range for each parameter of Ƥ was specified, for example, the number of RF trees, was varied between 1 and 150. Afterwards, all parameters of Ƥ were set to the start values, forming a set Ƥ’. Then the RF was trained on the training set with 25 datasets, using Ƥ’. In a subsequent validation step, the RF was used to segment the 4 datasets of the validation set and the resulting segmentation was compared to the gold standard using the Dice ratio as similarity metric. In the end, one of the parameters of Ƥ’ was changed to the next value in the specified range, thus forming a new Ƥ´, with which the RF was trained and validated again, till all possible parameter permutation had been tested.

The training result for each parameter set Ƥ’ was described by the Dice ratio D:

3 $$ D=\frac{2\left(\left|R\right|\cap \left|M\right|\right)}{\left|R+M\right|} $$

It quantified the percentage of overlap between the tested RF segmentation of the 4 validation datasets (R) and the corresponding manual segmentation (M). The training finished with picking the parameter set Ƥ’ with the highest average value of the Dice ratio. In our case, a Dice ratio difference of 0.1% meant that on average about 500 voxels were classified differently.

#### Parameter value ranges

The optimal number of trees was determined by CV-7 using a range of 1 to 150 trees. Similar, the samples per decision node was varied from 1 to 0.00001% of the total number of variables, which in our case resulted in 125,371 to 1 feature-label pairs.

Input parameters for Gabor filters (Ƥ_GF_) were size of the 2D Gabor kernel (given by a pixel window of *S*_*x*_ x *S*_*y*_), wavelength of the sinusoidal wave and standard deviation of the Gaussian. The following parameter ranges were used: Kernel size: 3 × 3 to 26 × 26 pixel window dimension; wavelength: 0.01 mm to 4 mm; standard deviation 0.01 mm to 4 mm.

Parameters for ELBP (Ƥ_ELBP_) were circle radius, number of samples on the circle and radius of the second, smaller circle for the spatial relationship. The radius of the second circle was empirically set to half the radius of the first one. CV-4 was used with the following ranges: Radius of the first circle: 0.5 to 5.5; number of samples: 4 to 28.

### Segmentation workflow

The trained RF was used to segment all 76 datasets. The segmentation workflow (Fig. 2b) is described in more detail in the following subsections.

#### Pre processing of T1 scans

MR images are often distorted by bias fields, caused by inhomogeneous magnetic fields of the coils. These image distortions were corrected in a pre processing step using the N4ITK algorithm [21], which is an improvement of the well-known and established N3 (nonparametric nonuniform intensity normalization) approach [22].

#### Segmentation of hand cross sectional area

The segmentation of the hand CSA was performed by a succession of basic image processing methods: first a threshold was used to roughly divide the T1 weighted image into background and hand. After N4ITK normalization described above, background intensities were around 10 and hand intensities around 500 units. For the particular scanner and MR acquisition protocol used in the study, a threshold of 70 was used. This resulted in an image containing one or more volumes of interests (VOI): one large volume of the hand and, due to image acquisition artifacts, multiple small volumes outside the hand VOI, where intensities were also higher than 70. These VOIs were smoothed by a 3D morphological opening. Finally, the hand VOI, which was always the largest VOI, was extracted.

#### Hand muscle segmentation

For segmentation, the features described above were calculated for each voxel using the optimal parameters Ƥ_GF_ and Ƥ_ELBP_ determined by the RF training. The trained RF (using Ƥ_RF_ as determined by the RF training) classifies each voxel into muscle and background, leading to a raw (i.e. without post processing) muscle segmentation.

#### Post processing of muscle segmentation

**Fig. 4:**
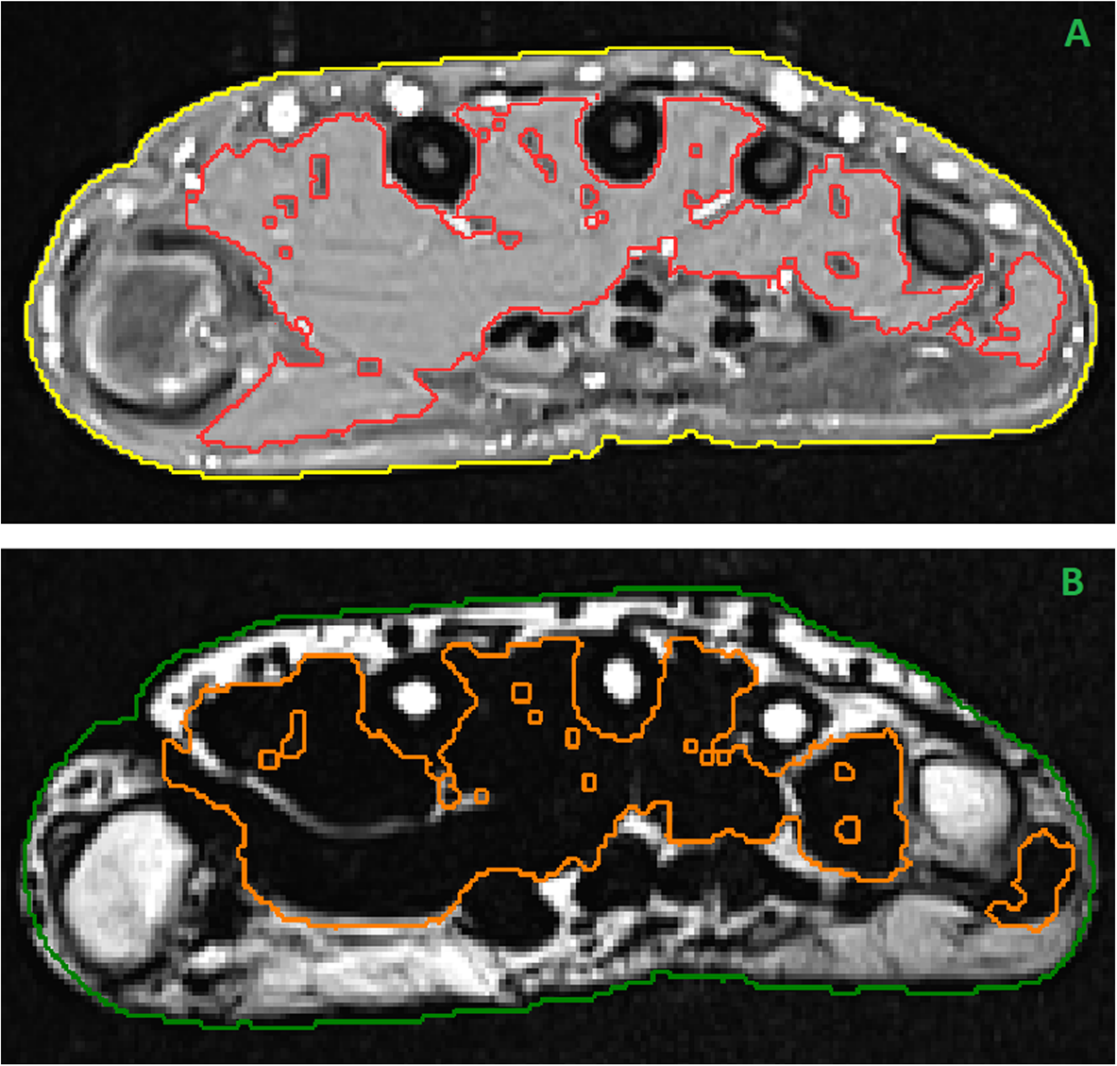
**a** Automated CSA (yellow) and muscle segmentation (red). This serves as input for the review and manual correction step by the clinical expert. **b** CSA (green) and muscle segmentation (orange) transferred to the Dixon image via rigid registration

The raw segmentation was post processed by a morphological dilation with radius 1, followed by island extraction, where islands (directly connected muscle voxels) with a size smaller than 10 voxels were discarded. Finally, a Gaussian function f(μ,σ) was fitted to the grey value histogram of the muscle segmentation mask and voxels with grey values outside the range [μ ± 3σ] were excluded from the segmentation. The underlying idea of this procedure was to include a wider area of voxels into the segmentation mask and then remove wrongly included voxels by the Gaussian fit. The result of the automated procedure is shown in Fig. 4a. The resulting muscle segmentation was reviewed by a clinical expert and manually edited if necessary.

#### Volume of interests

The volume of interest (VOIs) are hand volume V_H_ and muscle volume V_M_, obtained from the hand CSA and the muscle segmentation mask, respectively. In order to increase their longitudinal and cross sectional comparability, these two VOIs were manually limited to the metacarpal region defined by the metacarpal bone (MCP) III. For this purpose, the clinical expert had to set the proximal and distal MCP III boundaries. This could be achieved by navigating to the corresponding two slices in a transversal view.

#### Fat quantification

Fat was quantified using the Dixon fat fraction image. Since muscle was difficult to detect in the FF image, the muscle segmentation mask obtained using the T1 scans was transferred to the FF image via multimodal rigid image registration (Fig. 4b). The used similarity metric was mutual information as described by Mattes et al. [23], optimized by the gradient descent method. In the segmentation VOI the average and the absolute fat content was calculated.

### Validation of accuracy

Accuracy of the RF based segmentation was determined using the 30 gold standard datasets. Segmentation masks were compared between the manual and the RF approach using three different image metrics: the Dice ratio (Eq. 3), the average surface distance (Eq. 4) and the Hausdorff distance (Eq. 5). The average surface distance is the average of the distances from all points of one to the corresponding closest point of the other surface:

4 $$ {d}_{avg}=\frac{1}{\left|A\right|}\sum \limits_{a\in A}\underset{b\in B}{\mathit{\min}}\left\{d\left(a,b\right)\right\} $$

For d the Euclidean metric was used. The Hausdorff distance h is the maximum of the individual distances, i.e. the maximum local distance between the two segmentation masks:

5 $$ h=\underset{a\in A}{\mathit{\max}}\left\{\underset{b\in B}{\mathit{\min}}\left\{d\left(a,b\right)\right\}\right\} $$

### Reanalysis precision

For the determination of reanalysis precision errors, three operators analyzed 14 random data sets once (interoperator) and one operator analyzed the same 14 data sets three times (intraoperator). Reanalysis precision errors were calculated as root mean square average of standard deviation (RMS_SD) and coefficient of variation (CV) of individual data sets [24]. Precision was calculated for the hand segmentation in the T1 weighted scans, which depended on the manual determination of the MCP III length and potential manual segmentation corrections. Additionally the precision of the registration based fat quantification was calculated.

### Implementation details

The method was embedded in the Medical Image Analysis Framework (MIAF, Institute of Medical Physics, Erlangen, Germany). Implementation was done in C++ with the help of the Insight Segmentation and Registration Toolkit (ITK [25]) and the Open Source Computer Vision library (openCV [26]). For the analysis, a computer with a 3.4 GHz quadcore processor and 16 GB RAM was used.

## Results

### Random forest training

The manual hand muscle segmentation used as gold standard took 2.5 h ± 0.5 h per dataset. Random forest training for each parameter set Ƥ’ took between 15 min (for a RF with 7 trees) and 4 h (for a RF with 150 trees). The processing time mainly depended on the RF tree count. Since no user interaction was needed, the training was fully automated with an output of the Dice ratio for the individual parameter sets to file. The optimal parameter set Ƥ leading to the highest Dice ratio of 96% are summarized in Table 2.

**Table 2:** Optimal parameter set Ƥ as determined by RF training

RF number of trees	30
RF maximal tree depth	30
RF samples per node	0.0001%
GF kernel size	21 × 21
GF Gaussian sigma	1
GF Sinusoidal wavelength	1
ELBP (inner) radius	3 (1.5)
ELBP sample	8

Optimal parameter set Ƥ, showing the highest average Dice ratio during the validation step of the RF training

### Hand muscle segmentation

The N4ITK pre-processing and the hand muscle segmentation was automated and did not need any user input. Pre-processing took 30 s ± 10 s. The segmentation took 2 min ± 0.2 min, of which the RF based part described required about 90% of the time.

### User interaction

**Fig. 5:**
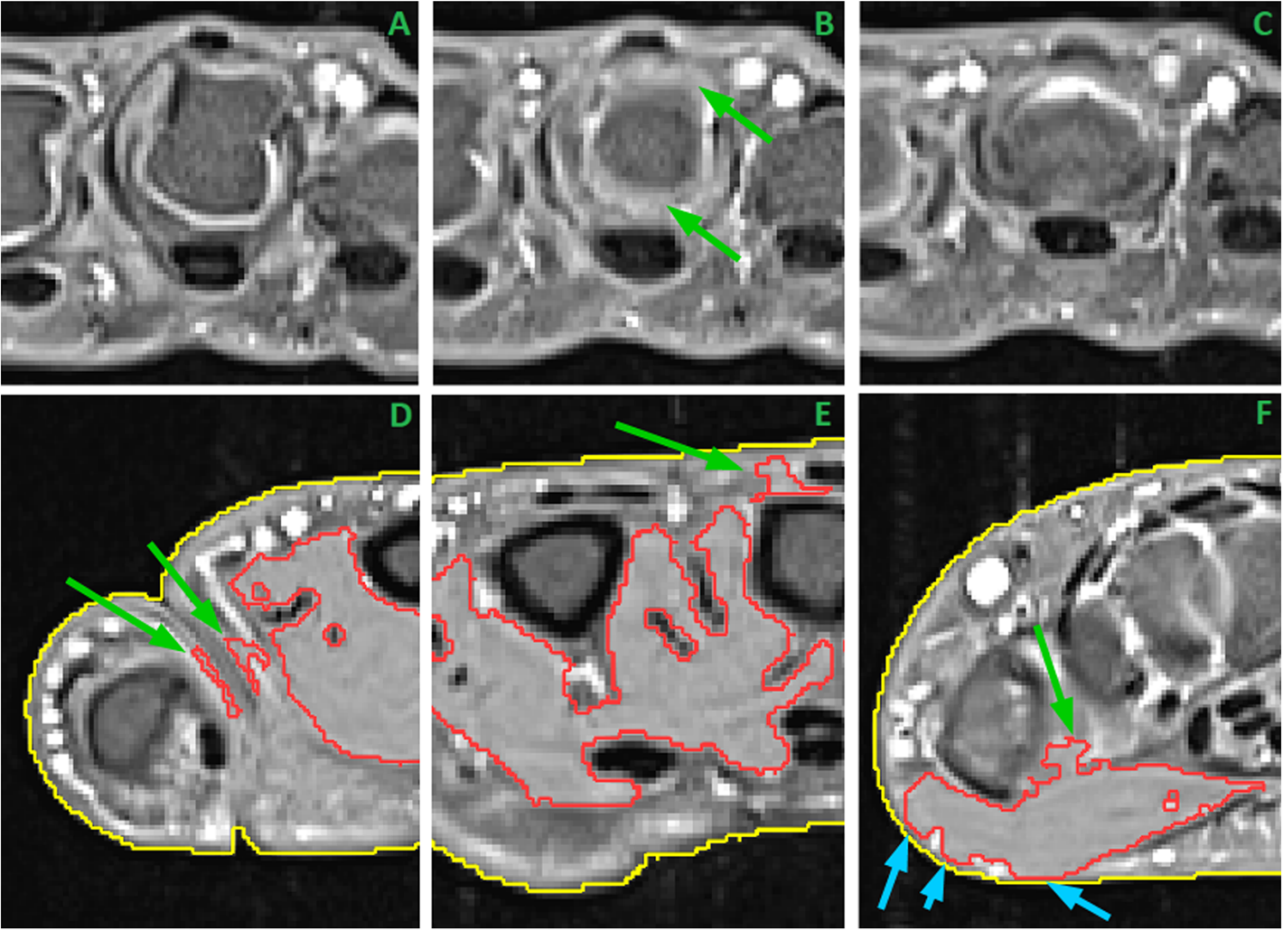
The top row shows the three transversal slices of the distal MCP III joint: **a** with characteristic rectangular MCP III shape, **b** with round joint shape and synovial fluid (green arrows) and **c** with shape of proximal phalange. The bottom row shows three common classification errors, which required manual correction: **d** shows segmented skin layers between MCP I and II (green arrows), **e** shows segmented subcutaneous areas close to the MCP III bone (green arrow) and **f** shows leaking segmentation into joint area (green arrow) and skin (light blue arrows)

The user interaction for determining the MCP area could be completed in less than 30s. Manual segmentation editing was necessary in 69 of the 76 datasets. This was mostly limited to deleting segmented forearm muscles or thicker layers of skin and was not considered tedious by the operator (see Fig. 5 bottom row). The manual editing per dataset took 7 min ± 5 min. Multimodal registration to Dixon sequence took 1 min on average. The registration quality was visually checked; no dataset needed further adjustments. All 76 datasets could be analyzed conveniently and the results were considered appropriate, as judged by the medical expert.

### Accuracy

Accuracy results based on the comparison to the 30 gold standard datasets are listed in Table 3.

**Table 3:** Accuracy results

*n* = 30	Mean	SD	Min	Max
D	0.9810	0.0442	0.8937	0.9998
d_avg_ [mm]	0.0400	0.104	0.0010	0.4428
h [mm]	1.094	1.610	0.0324	3.76

*Abbreviations*: *SD* standard deviation, *D* dice ratio, *d*_*avg*_ average surface distance, *h* Hausdorff distance

High Hausdorff distance values were observed in scanning direction at the proximal and distal ends of the analyzed VOIs. A closer inspection showed, that high Hausdorff distances were caused by variations of the manual placements of the MCP III borders to define the analysis VOIs.

### Reanalysis precision

Inter- and intraoperator reanalysis precision errors for muscle segmentation and fat quantification are summarized in Table 4.

**Table 4:** Inter- and intraoperator reanalysis precision errors

	Hand volume		Muscle volume	
Interoperator	5.0 mm^3^	0.19%	1.5 mm^3^	0.24%
Intraoperator	3.4 mm^3^	0.13%	0.35 mm^3^	0.05%
	Fat content		Fat fraction	
Interoperator	364.4 mm^3^	0.60%	0.09%	0.07%
Intraoperator	41.6 mm^3^	0.10%	0.006%	0.04%
	MCP III length			
Interoperator	0.98 mm	0.12%		
Intraoperator	1.2 mm	0.15%		

Inter- and intraoperator reanalysis precision errors of hand and muscle volume, fat quantification and MCP III length. Precision errors are given as root mean square averages of standard deviations and coefficients of variation

## Discussion

We presented a novel, highly accurate and precise segmentation method for hand muscles in T1 weighted fat suppressed MR scans, which was based on Random Forest classifiers. Muscle fat quantification was measured after multi-modal image registration from T1 weighted to 2-pt Dixon sequences. The two main outcomes of the described method were the large reduction of required user interaction time per dataset and a high accuracy and reanalysis precision. The clinical relevance may be high, since this approach provides a realistic perspective to integrate quantitative muscle assessments in research and clinical routine. Such studies can provide new insight of the interaction between RA and muscle. Examples are the relation between the inflammatory potential of intramuscular adipose tissue with inflammatory pathways of RA, the pathophysiological mechanisms of hand muscle strength training by physiotherapy and exercise, the effect of pharmaceuticals like drug-modifying anti-rheumatic drugs (DMARDs) on the hand muscle, or the comparison of RA and age related muscle degenerative processes.

The hand comprises many different tissues, partitioned into compartments of different size and shape. Separating muscle from these tissues by manual outlining is a tedious and very time-consuming task, often impeding or even prohibiting even smaller studies. Image processing methods can reduce the analysis time tremendously. The method developed in this paper proposes an automated muscle segmentation to the user, most often a rheumatologist, for review and optional manual editing. Its aim was not a substitution of a clinical expert reviewer, but to decrease interaction time. The overall manual interaction time per dataset could be reduced from about 2.5 h (gold standard muscle segmentation) to about 15 min per dataset.

The identification of the poximal and distal delimiting transversal slices of the MCP III bone was straightforward, because the joint areas showed a characteristic pattern in the transversal view. For example Fig. 5 top row shows the three transversal slices covering the distal MCP III joint. Panel A shows the characteristic rectangular shape of the distal MCP III bone. Panel C shows the first slice of the proximal phalange, while panel B displays the transversal slice through the joint in between, with visible synovial fluid. In the majority of cases segmentation correction was limited to removal of parts which were wrongly classified as muscle, since they showed similar intensity characteristics. Figure 5 bottom row shows three common examples: panel D shows the area between MCP I (thumb) and MCP II (index finger), in which layers of skin were wrongly classified as muscle. In panel E, subcutaneous areas above the MCP III bone were included into the muscle segmentation. Panel F shows the thenar segmentation in the most proximal slice of the muscle VOI. The segmentation in this slice was leaking into inter-bone areas and subcutaneous layers.

Accuracy of the RF segmentation was excellent. The average surface distance (0.04 mm) was low, and about one magnitude smaller than the in-plane voxel size (0.5 mm). The average Dice ratio was high (98%). The maximum Hausdorff distances were observed in scanning direction at the proximal and distal ends of the analyzed VOIs. Given the image slice thickness of 3 mm, the higher values for h corresponded to about one voxel in slice direction. It turned out, that for those scans the analysis VOI between the gold standard and RF based segmentation was shifted by one slice in scan direction. On the other hand, this did not affect D or d_avg,_ since the muscle volumes in the boundary slices are very small compared to the total muscle volume. As the proximal and distal border of the analysis VOI are selected by the operator, an automation of this step will likely further decrease Hausdorff distances.

Reanalysis precision of the segmentation was excellent. As expected, intraoperator was better than interoperator precision. Interestingly, intraoperator precision for hand volume was worse than for muscle volume. Thus, variations of the operator defined MCP III length affect hand more than muscle volume. Surprisingly, this precision is worse for intraoperator compared to interoperator. Nevertheless, the RMS_SD of the MCP III lengths is much smaller than the slice thickness of 3.3 mm. Thus, the user interaction did not have major impact on the analysis precision.

The interaction was limited to removal of skin and subcutaneous parts, which were clearly distinguishable from the muscle. Thus it could be done without altering the main muscle segmentation. Reason for these wrongly classified parts are the similar mean grey value and their deviation and texture, compared to the muscle tissue. Reanalysis precision of the fat quantification via registration was excellent. Interoperator precision of the total fat content (0.6%) was slightly higher than the other measured variations. Small inaccuracies of the registration to the Dixon sequence may lead to the inclusion of e.g. subcutaneous fat, explaining the higher precision error. Since the total muscle volume is big enough, the precision of the fat fraction measurement is still excellent.

The method was based on datasets of RA patients only. This would be a limitation to address clinical questions as for example raised in the introduction. For a clinical study also the sample size were small. However, in this study an efficient segmentation method to be used in a clinical study was proposed. For this purpose a control group of healthy subjects adds little value. A method based on scans from RA patients with impaired muscle seems to be well generalizable to healthy subjects. Since the inflammation in RA patients is mainly located in and around the synovial joints, which are not part of the segmentation, the inflammation will to the best of our knowledge not influence the measurement. Furthermore, the used cohort of outclinic patients spans a wide age range and a large range of disease duration, making it an ideal candidate for developing such a method. For fat quantification, an additional MR Dixon sequence was added to the standard clinical hand protocol.

With respect to segmentation, a fuzzy clustering based approach like in [13] was discarded, because the grey value histogram of the hand could not be reliably partitioned into separate clusters. Even more elaborate ideas in [14] were not considered, because a distinction between different hand muscle groups, e.g. thenar and hypo-thenar, was not the topic of the current study. Another idea tested was based on a multi atlas: the 30 manually segmented gold standard MR scans were combined to a multi atlas. New segmentations were generated by registration of each atlas to the new image. However this approach was abandoned due to the high shape variability of the hand during the scans. Thus, methods incorporating shape information were not further considered.

## Conclusions

We have developed a new highly precise method to segment hand muscle. In combination with quantitative fat measurements obtained from Dixon sequences the intramuscular fat content can be determined. This is a prerequisite for further studies to investigate and quantify the impact of muscle fat infiltration on RA and vice versa. Especially the question if obesity is associated with RA or even accelerates RA progress can now addressed as a larger amount of data can analyzed in a reasonable time.

## Data Availability

The datasets generated and/or analysed during the current study are not publicly available since the patient consent form did not include such agreement, but are available from the corresponding author on reasonable request.

## Abbreviations

BMI: Body mass index
CRP: C-reactive protein
CSA: Cross sectional area
CV: Coefficient of variation
CV-x: x-fold cross validation
D: Dice ratio
DAS28: Eular disease activity score 28
d_avg_: Average surface distance
ELBP: Extended local binary pattern
ESR: Erythrocyte sedimentation rate
FF: Fat fraction
h: Hausdorff distance
MCP: Metacarpal bone
MR: Magnetic resonance
N3: Nonparametric nonuniform intensity normalization
RA: Rheumatoid arthritis
RF: Random forest
RMS_SD: Root mean square average of standard deviation
SAT: Subcutaneous adipose tissue
SD: Standard deviation
TSE: Turbo spin echo
VOI: Volume of interest
